# Fronto-striatal gray matter contributions to discrimination learning in Parkinson's disease

**DOI:** 10.3389/fncom.2013.00180

**Published:** 2013-12-12

**Authors:** Claire O'Callaghan, Ahmed A. Moustafa, Sanne de Wit, James M. Shine, Trevor W. Robbins, Simon J. G. Lewis, Michael Hornberger

**Affiliations:** ^1^Neuroscience Research AustraliaSydney, NSW, Australia; ^2^Faculty of Medicine, School of Medical Sciences, University of New South WalesSydney, NSW, Australia; ^3^School of Social Sciences and Psychology and the Marcs Institute for Brain and Behaviour, University of Western SydneySydney, NSW, Australia; ^4^Cognitive Science Center Amsterdam and Department of Clinical Psychology, University of AmsterdamAmsterdam, Netherlands; ^5^Parkinson's Disease Clinic, Brain and Mind Research Institute, University of SydneySydney, NSW, Australia; ^6^Department of Psychology, Behavioural and Clinical Neuroscience Institute, University of CambridgeCambridge, UK; ^7^ARC Centre of Excellence in Cognition and its DisordersSydney, NSW, Australia; ^8^Department of Clinical Neurosciences, University of CambridgeCambridge, UK

**Keywords:** Parkinson's disease, discrimination learning, goal-directed learning, computational modeling, voxel-based morphometry, fronto-striatal

## Abstract

Discrimination learning deficits in Parkinson's disease (PD) have been well-established. Using both behavioral patient studies and computational approaches, these deficits have typically been attributed to dopamine imbalance across the basal ganglia. However, this explanation of impaired learning in PD does not account for the possible contribution of other pathological changes that occur in the disease process, importantly including gray matter loss. To address this gap in the literature, the current study explored the relationship between fronto-striatal gray matter atrophy and learning in PD. We employed a discrimination learning task and computational modeling in order to assess learning rates in non-demented PD patients. Behaviorally, we confirmed that learning rates were reduced in patients relative to controls. Furthermore, voxel-based morphometry imaging analysis demonstrated that this learning impairment was directly related to gray matter loss in discrete fronto-striatal regions (specifically, the ventromedial prefrontal cortex, inferior frontal gyrus and nucleus accumbens). These findings suggest that dopaminergic imbalance may not be the sole determinant of discrimination learning deficits in PD, and highlight the importance of factoring in the broader pathological changes when constructing models of learning in PD.

## Introduction

Parkinson's disease (PD) is a neurodegenerative condition characterized by hallmark motor disturbances, with its primary neuropathology in the nigrostriatal pathway. This leads to severe dopamine depletion in the dorsal striatum, while the ventral striatum is relatively preserved in the earlier disease stages (Jellinger, 2001). In PD, both the progressive dopamine depletion in the basal ganglia and the concurrent beneficial and deleterious effects of dopamine replacement medications, have been associated with a range of distinct learning impairments (for reviews, see Price et al., 2009; Foerde and Shohamy, 2011b). These dopamine dependent learning deficits in PD have been informative in the development of theoretical accounts of learning function and have provided important advances and testable predictions for computational explanations of learning (Frank, 2005). In particular, PD has been associated with acquisition deficits in feedback-based discrimination learning (Myers et al., 2003; de Wit et al., 2011), which have also been described via computational approaches (Moustafa et al., 2010).

Feedback-based and trial-and-error learning is presumed to be mediated by relative patterns of tonic vs. phasic dopamine activity occurring in response to environmental reinforcers (Schultz, 2002; Bromberg-Martin et al., 2010). Indeed, current accounts of discrimination learning in PD have been derived through ON- vs. OFF-medication patient studies and through computational models, which have established a role for basal ganglia dopamine imbalance as a crucial factor underpinning the feedback-based learning deficits (Frank et al., 2004; Shohamy et al., 2006). Whilst such explanations of learning deficits based on dopaminergic imbalance do accord with the biological characteristics of PD, these theories have not addressed the potential contributions of other prevalent pathological effects in PD. For example, in addition to the characteristic dopamine depletion PD is also associated with gray matter loss and reduced white matter integrity (Duncan et al., 2013). Significantly, regions of gray matter loss in PD involve systems that are implicated in a range of higher level cognitive functions (including learning), and it is only more recently that direct associations between volumetric reductions and specific cognitive deficits have been confirmed in early stage, non-demented PD (Filoteo et al., 2013; O'Callaghan et al., 2013).

Given the known volumetric brain changes in PD and the possibility that they may directly affect learning processes, exploring this relationship to inform future learning theories and computational approaches that rely on PD as a model is now vital. In the current study, we directly examined this issue by combining voxel-based morphometry analysis with a computational modeling technique in order to determine how fronto-striatal gray matter reductions relate to acquisition efficiency on a discrimination learning task. We hypothesized PD patients would show impaired learning acquisition rates and that these impairments would be associated with volumetric reductions in fronto-striatal regions that are crucial for feedback-based learning and reward processing.

## Materials and methods

### Case selection

Seventeen non-demented PD patients were recruited from the Brain and Mind Institute Parkinson's Disease Research Clinic; all satisfied UKPDS Brain Bank criteria for diagnosis of PD (Gibb and Lees, 1988) and were between Hoehn and Yahr stages I and III (Hoehn and Yahr, 1967). Motor score from the Unified Parkinson's Disease Rating Scale (UPDRS-III) (Goetz et al., 2008) is also reported. One patient was untreated; three were on levodopa monotherapy and two were taking levodopa plus an adjuvant; nine patients were on levodopa plus a dopamine agonist, and in this group four were also taking an adjuvant and one was taking a monoamine oxidase inhibitor; one patient was on a dopamine agonist plus a monoamine oxidase inhibitor and one was taking a monoamine oxidase inhibitor only. Treated patients performed behavioral testing in the ON state, having taken their usual medications. L-dopa daily dose equivalents (DDE mg/day) were calculated for treated patients. Patients with overt clinical depression were not included in the study and a measure of affective disturbance was obtained (Beck Depression Inventory-II; BDI-II, Beck et al., 1996). Eleven age- and education-matched healthy controls were selected from a volunteer panel. See Table 1 for demographic details and clinical characteristics.

**Table 1:** **Mean (SD) values for Controls and PD patients on demographics, clinical characteristics and discrimination learning measures**.

**Demographics, clinical characteristics and executive function**	**Controls**	**PD**	***F*/χ 2-values**
N	11	17	–
Sex (M:F)	3:8	13:4	–
Age (years)	66.3 (7.2)	66.4 (8.4)	n.s.
Education (years)^a^	14.9 (2.0)	14.1 (3.6)	n.s.
MMSE (max. 30)^a^	29.6 (0.71)	28.6 (1.6)	n.s.
Disease duration (years since diagnosis)	–	5.6 (5.4)	–
Hoehn and Yahr stage	–	2.1 (0.52)	–
UPDRS III	–	29.2 (12.8)	–
Dopamine dose equivalent (mg/day)	–	616.1 (453.1)	–
BDI-II	–	10.6 (6.9)	–
**EXECUTIVE FUNCTION**			
Digit span forwards	11.4 (1.8)	10.9 (2.3)	n.s.
Digit span backwards	9.1 (2.1)	7.4 (1.8)	*
Letter fluency	49.0 (15.9)	41.2 (13.5)	n.s.
Trail making test B-A	24.5 (19.4)	41.8 (25.0)	n.s.
**DISCRIMINATION LEARNING**			
Overall accuracy (%)	82.3 (10.6)	71.9 (19.6)	n.s.
Learning rate	0.217 (0.036)	0.163 (0.041)	**
Exploration^a^	0.70 (0.26)	0.85 (0.30)	n.s.

n.s., non significant,

* p < 0.05,

** p < 0.001; F-values indicate significant differences across groups, otherwise due to unequal variance χ2 indicates differences across groups^a^. MMSE, Mini-Mental State Examination; UPDRS III, Motor score from the Unified Parkinson's Disease Rating Scale; BDI-II, Beck Depression Inventory II.

The research study was approved by the Human Ethics Committees of the Central and South Eastern Sydney Area Health Services and the Universities of Sydney and New South Wales, and complies with the statement on human experimentation issued by the National Health and Medical Research Council of Australia.

### Neuropsychological assessment

All patients and controls were administered the Mini Mental State Examination (MMSE; Folstein et al., 1975) to determine their overall cognitive functioning. For detailed measurement of executive function, patients and controls underwent a battery of tests including Verbal Fluency [measured by the number of words produced in 60 s, beginning with F, A, and S (Benton et al., 1994)]; the Trail-Making test (time B-A) to assess speeded set-shifting (Reitan and Wolfson, 1985); and a Digit Span task, with digits repeated in their original order (forwards) and in reverse order (backwards) (Wechsler, 1997) to assess attention span and working memory.

### Discrimination learning task

We administered a discrimination learning task developed by de Wit and colleagues, which was an abbreviated version of a more extensive instrumental learning measure described by de Wit et al. (2007). The task was computer based and programmed using Visual Basic 6.0, with keyboard response keys *z* and *m* programmed to register a *left* or *right* response.

Discrimination learning tasks involve a discriminative stimulus that signals whether or not a certain response will lead to a particular outcome; stimuli are presumed to have acquired discriminative control over instrumental performance when correct responding occurs in the presence of a given stimulus (i.e., when the stimulus: response-outcome contingency is acquired) (Bouton, 2007). In the current discrimination learning task, for each trial the discriminative stimulus consisted of a colored icon depicting a piece of a fruit on the front of a box. There were six possible fruits that could be pictured on the outside of the box (i.e., strawberry, lemon, grape, kiwi, melon, and orange). Subjects were required to make either a *left* or *right* response in order to “open” the box and obtain the outcome/reward inside (the outcome being a different fruit, i.e., coconut, pear, pineapple, cherry, banana, and apple). Each of the six stimulus fruits were associated with a particular correct response (i.e., *left* or *right*) that would result in obtaining the reward/outcome. These contingencies were kept constant, for example a *left* response to the strawberry stimulus would always result in the box opening to reveal an outcome/reward, whereas if a *right* response was made to the strawberry stimulus, the box would open to reveal nothing inside. Additional feedback was provided as the opened box revealing the reward was paired with a positive sound and points displayed on the screen, whereas the opened box with nothing inside was paired with a negative sound effect. The initial fruit stimulus remained on the screen until subjects made a response and faster correct responses earned more points (in the range from 1 to 5). The outcome fruit was presented for 1 s, and inter-trial intervals were fixed at 1.5 s.

Subjects were instructed at the outset of the task that they would need to determine the correct response for each stimulus fruit via a trial and error process. It was emphasized that these contingencies would not change throughout the trials, so that it would be possible for them to learn these stimulus-response associations. They were also encouraged to memorise the stimulus: response-outcome associations, as they would be questioned on them at the end.

Each subject completed 96 trials, comprising of eight 12-trial blocks during which each of the six possible stimulus-response pairs was presented twice in a randomized order; three of the stimulus fruits were associated with a correct *left* response and the other three were associated with a correct *right* response. Across subjects, the particular fruits that served as the stimulus and those that served as the outcome were counterbalanced. From the discrimination learning task, we derived a binary outcome measure of either 1 or 0 for each trial (1 indicating a correct response for that trial, 0 an incorrect response). Finally, after completing the trials, patients were asked to fill in pencil and paper questionnaires that probed explicit knowledge of the stimulus: response-outcome contingencies. These questionnaires were divided into three parts (each with six items), assessing knowledge of: (1) stimulus-response knowledge; (2) response-outcome; and (3) stimulus-outcome. In part (1), subjects were shown pictures of each stimulus fruit one at a time and they were asked to verbally indicate whether a *left* or *right* response was associated with obtaining a reward for each stimulus. A similar procedure was followed in part (2), as subjects were shown each reward/outcome and asked to indicate whether a *left* or *right* response had been necessary to successfully achieve that reward. In part (3), subjects were shown each stimulus fruit alongside an array of all possible reward fruits and they selected the reward that had been paired with each particular stimulus.

### Computational model

Given the insufficiency of classical statistical methods in extracting learning rates and trial-by-trial responses, we applied the reinforcement Q-learning model to the outcome measures generated from the discrimination learning task, for each subject's pattern of correct and incorrect responses across the 96 trials (Sutton and Barto, 1998). The input of this model is a trial-by-trial sequence of responses for each subject, while the output is the learning rate and exploration parameter values, which cannot be obtained from regular statistical analysis of behavioral data. Previous research has used similar computational models to fit model parameter values for each subject in genetic (Frank et al., 2007) and patient studies (Gold et al., 2012). The rationale for applying the Q-learning model to the behavioral data is to disentangle each subject's performance to different components, and also to determine which model parameters can better account for variations in behavioral performance across different groups. Here, we attempt to understand the observed behavioral results using the computational reinforcement Q-learning model (Watkins and Dayan, 1992; Sutton and Barto, 1998; Frank et al., 2007) and specifically, we have fitted our behavioral data using a Q-learning model (Frank et al., 2007).

By using the reinforcement Q-learning model, we fit individual subject's trial-by-trial data, which culminates in two parameter values that correspond to the subject's learning rate and exploration/exploitation bias. The learning rate parameter modulates the degree to which feedback on the current trial is used to adjust expectations for future trials. The exploration parameter indicates whether the subject is more likely to choose the same or a different response as on previous trials with the same stimulus. A small exploration/exploitation parameter indicates exploitation (i.e., increased likelihood that subjects will choose the same response as previously made, when presented with the same stimulus), and a large value indicates exploration (i.e., increased likelihood they will choose a different response when presented with the same stimulus). In principle, impaired feedback learning can occur because of small learning rate or decreased likelihood to explore alternative responses at the expense of exploiting previously erroneous response strategies.

Specifically, we compute a weight (*W)* value for selecting each stimulus *i* during trial *t*, such that the value of the chosen stimulus is modified by reinforcement feedback:

PE(t)=US(t)−W(t)

**w**here *PE*(*t*) is the prediction error at time t; *US*(*t*) is feedback presented at time t, and is equal to 1 for positive and 0 for negative feedback. *W*-values are computed using the following equation.

$$W_{i}(t+1)=W_{i}(t)+\alpha PE(t)$$

where α is learning rate (for more details, see Frank et al., 2007).

We have modeled choice by using a softmax logistic function, with inverse gain (exploration) parameter β, such that the probability of choosing A over B was computed as:

$$P_{A}(t)=\frac{e^{W_{A}(t)/\beta }}{e^{W_{A}(t)/\beta }+e^{W_{B}(t)/\beta }}$$

Each participant's trial-by-trial choices were fitted with two free parameters, α and β, which were selected to maximize fit to participant's sequence of choices in the task. β is an inverse gain parameter and reflects the participant's tendency to either exploit (i.e., to choose the response with the currently highest *W*-value) or explore (i.e., to randomly choose a category).

We then fitted the model to each participant's data, by searching through the space of each of these two parameters from 0 to 1 with a step size of 0.01. We then optimized the log likelihood estimate (LLE) at trial t:

$$\text{LLE}=\text{Log }(\Pi_{t}P(t))$$

where t is trial number (for a total of 96 trials). For each participant, the best fitting parameter values are those associated with maximum LLE. Equivalently, maximum LLE is the most predictive of the participant's responses in the task. In this model, the best fitting parameter values to each participant's behavioral data accommodate trial-by-trial adaptations in response to feedback given based on participants' choices. In addition, we predict that these values will explain differences in learning efficiency between patients and controls.

Finally, to validate our model we compared our results with a random responder model. Specifically, we calculated the pseudo-*R*^2^ measure, which is (LLE-*r*)/*r*, where *r* is the log likelihood of the data under a model of purely random choices, in which *p* = 0.5 for all trials (Camerer and Ho, 1999; Daw et al., 2006). The resulting pseudo-*R*^2^ statistic reveals how well the model fits the data compared to a model predicting chance performance and is independent of the number of trials to be fit in each set (see Frank et al., 2007, for discussion).

### Behavioral analyses

Data were analyzed using SPSS19.0 (SPSS Inc., Chicago, Ill., USA). Parametric demographic and neuropsychological data were compared across the groups via One-Way ANOVAs followed by Tukey *post-hoc* tests. A priori, demographic and learning variables were plotted and checked for normality of distribution by Kolmogorov-Smirnov tests. Variables showing non-parametric distribution were analyzed via Chi-square, Kruskal-Wallis and Mann-Whitney *U*-tests. A repeated measures ANOVA with Bonferroni *post hoc* tests was used to explore group differences in learning accuracy across the eight blocks, with group (control vs. patient) as the between-subjects variable and block (blocks 1–8) as the within-subjects variable.

### Imaging acquisition

All patients and controls underwent the same imaging protocol with whole-brain T1 images acquired using 3T Philips MRI scanners with standard quadrature head coil (8 channels). The 3D T1-weighted sequences were acquired as follows: coronal orientation, matrix 256 × 256, 200 slices, 1 × 1 mm^2^ in-plane resolution, slice thickness 1 mm, TE/TR = 2.6/5.8 ms.

### Voxel-based morphometry (VBM) analysis

3D T1-weighted sequences were analyzed with FSL-VBM, a voxel-based morphometry analysis (Ashburner and Friston, 2000; Good et al., 2001) which is part of the FSL software package http://www.fmrib.ox.ac.uk/fsl/fslvbm/index.html (Smith et al., 2004). First, tissue segmentation was carried out using FMRIB's Automatic Segmentation Tool (FAST) (Zhang et al., 2001) from brain extracted images. The resulting gray matter partial volume maps were then aligned to the Montreal Neurological Institute standard space (MNI152) using the non-linear registration approach using FNIRT (Andersson et al., 2007a,b), which uses a b-spline representation of the registration warp field (Rueckert et al., 1999). The registered partial volume maps were then modulated (to correct for local expansion or contraction) by dividing them by the Jacobian of the warp field. The modulated images were then smoothed with an isotropic Gaussian kernel with a standard deviation of 3 mm (FWHM: 8 mm). A region-of-interest (ROI) mask for prefrontal and striatal brain regions was created by using the Harvard-Oxford cortical and subcortical structural atlas. The atlas regions that comprise the entire prefrontal cortex and striatum were included in the mask, these included frontal pole, superior frontal gyrus, middle frontal gyrus, inferior frontal gyrus, frontal medial cortex, subcallosal cortex, paracingulate gyrus, cingulate gyrus (anterior division), frontal orbital cortex, caudate, putamen, and nucleus accumbens. Finally, a voxelwise general linear model (GLM) was applied and permutation-based non-parametric testing was used to form clusters with the Threshold-Free Cluster Enhancement (TFCE) method (Smith and Nichols, 2009), tested for significance at *p* < 0.05, corrected for multiple comparisons via Family-wise Error (FWE) correction across space, unless otherwise stated.

## Results

### Demographics, clinical characteristics and neuropsychological assessment

Demographics and general cognitive scores can be seen in Table 1. Participant groups did not differ in terms of age, education or MMSE score (*p*'s > 0.1). Patients and controls did not differ in their Digit Span forwards score (*p* > 0.6), but patients were impaired relative to Controls for Digit Span backwards (*p* < 0.05). Groups were equivalent for Letter Fluency scores (*p* > 0.2) and although groups did not differ significantly on Trail Making B-A scores, there was a strong trend toward worse performance in the patients (*p* = 0.06). See Table 1.

### Learning measures

**Figure 1:**
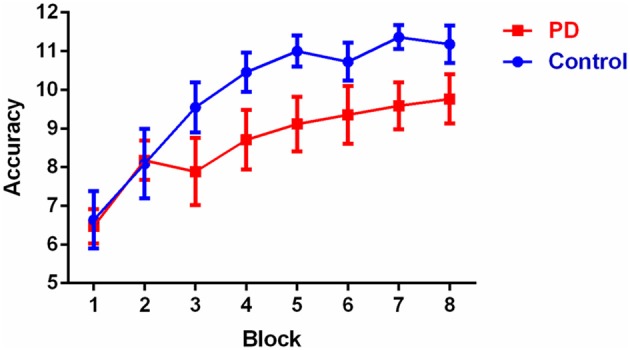
**Mean accuracy scores (with standard error bars) across the eight 12-trial blocks**.

Overall accuracy scores on the discrimination learning task are shown in Table 1 and learning accuracy across the eight blocks is shown in Figure 1. Overall accuracy across the 96 trials was not significantly different between the groups (*p* > 0.1). Results of the repeated measures ANOVA showed that there was no significant main effect of group [*F*_(1, 26)_ = 2.6, *p* > 0.1]. Mauchly's test indicated that the assumption of sphericity had been violated [χ^2^_(27)_ = 71.0, *p* < 0.001] therefore degrees of freedom were corrected using Huynh-Feldt estimates of sphericity (ε = 0.550). The results show a significant main effect for block [*F*_(4.8, 124.2)_ = 20.3, *p* < 0.001], which reflected that, irrespective of group, accuracy in blocks 6, 7, and 8 was significantly higher than in blocks 1, 2, and 3 (*p*-values < 0.05), accuracy in block 5 was significantly higher than in blocks 1 and 2 (*p* < 0.05), and accuracy in block 4 was higher than accuracy in block 1 (*p* < 0.002). There was no significant group by block interaction [*F*_(4.8, 124.2)_ = 1.6, *p* > 0.1). *Post-hoc* between-group comparisons revealed that controls and PD patients only differed significantly on their accuracy in block 7 with controls having a higher accuracy score (*p* < 0.05), no significant difference were observed in other blocks (*p* > 0.05). Within-group *post-hoc* analysis showed that controls had consistent significant differences in accuracy between early and late blocks, with blocks 4, 5, 6, 7, and 8 all having higher accuracy than both blocks 1 and 2 (*p*-values < 0.05). PD patients showed a slightly less consistent pattern, with accuracy in blocks 5, 6, 7, and 8 higher than in block 1 (but not block 2) (*p*-values < 0.05); with all other block accuracies were equivalent, expect for blocks 7 and 8 being significantly higher than block 3 (*p*-values < 0.05).

Results of the learning rate and exploration parameters for the discrimination learning task, as derived from the computational model, are also shown in Table 1. Exploration parameters did not differ significantly between the groups (*p* > 0.3) and the small value of the parameter in both patients and controls suggested minimal exploration, which would be predicted based on the nature of the task. Learning Rate for the PD patients was significantly reduced relative to controls (*p* = 0.001) and these Learning Rate values were further analyzed in the VBM analysis. Results from the random responder model revealed the mean and standard deviation of pseudo-*R*^2^ were 0.2901 and 0.173, respectively. This was significantly larger than zero, indicating our model performs better than chance at fitting individuals' data.

Participant groups did not differ in terms of explicit knowledge of Stimulus-Response-Outcome contingencies. The following mean (standard deviation) results on the three questionnaire sections were achieved, each section with a possible maximum score of 6 (i.e., 1 point per item). Stimulus-Response accuracy for controls was 5.6 (0.05) and for PD patients 5.3 (1.6); Response-Outcome accuracy for controls was 5.0 (1.2) and PD patients 4.6 (1.7); Stimulus-Outcome for controls was 3.5 (1.7) and PD patients 3.0 (2.2), with all *p*-values > 0.5. In a correlation analysis, none of the PD clinical variables (i.e., disease duration, Hoehn and Yahr stage, UPDRS III, DDE mg/day, BDI score) or the digits backward score, showed a significant relationship with the Learning Rate measure (*p*'s > 0.1).

### VBM analysis

The PD group was initially contrasted with controls to reveal overall patterns of brain atrophy in the fronto-striatal mask. PD patients showed gray matter atrophy bilaterally in the frontal orbital cortex and subcallosal cortex, extending back to the left ventral striatal (nucleus accumbens) territory; as well as in the inferior frontal gyri bilaterally (see Supplementary Table 1).

**Figure 2:**
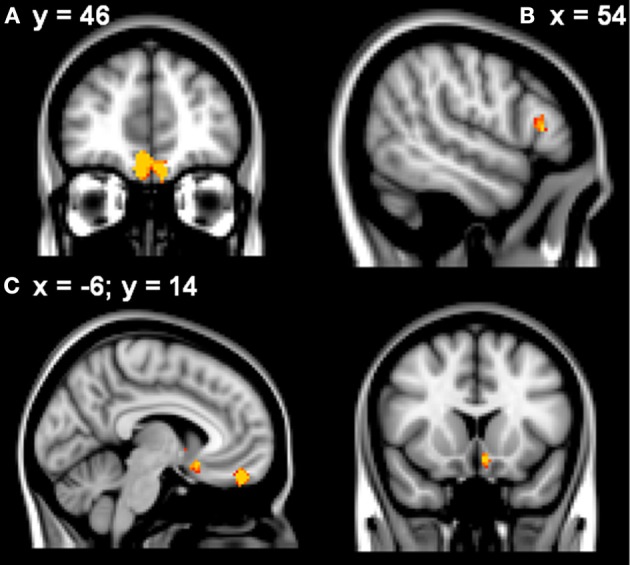
**VBM analysis showing the frontal and striatal regions that correlated with elevated learning rates in the patients in (A) frontal medial cortex (B) right inferior frontal gyrus (C) subcallosal/left nucleus accumbens**. Clusters are overlaid on the MNI standard brain (*t* > 2.50). Cultured voxels show regions which were significant in the analyses for *p* < 0.01 uncorrected and a cluster threshold of 40 contiguous voxels.

Learning rate was then entered as a covariate in the design matrix of the VBM analysis. For PD patients, Learning Rate score covaried with gray matter atrophy in the frontal medial cortex/frontal pole, the right inferior frontal gyrus and the left subcallosal cortex/left nucleus accumbens (see Table 2 and Figure 2).

**Table 2:** **Region of interest Voxel-based morphometry (VBM) results showing areas of significant gray matter intensity decrease that covary with learning measures**.

**Regions**	**Hemisphere (L/R/B)**	**MNI coordinates**			**Number of voxels**	***T*-score**
		***X***	***Y***	***Z***		
**LEARNING RATE**						
Frontal medial cortex; Frontal pole	B	−6	46	−26	422	2.70
Inferior frontal gyrus	R	54	26	8	54	
Subcallosal/extending back to L NAcc	L	−4	12	−14	46	

All results uncorrected at p < 0.01; only clusters with at least 40 contiguous voxels included.

Finally, a partial correlation analysis was used to explore whether common damage to the ventromedial prefrontal cortex, right inferior frontal gyrus and left subcallosal cortex/nucleus accumbens explained the significant correlations with Learning Rate. The ventromedial prefrontal region still correlated significantly with Learning Rate (*p* < 0.05) when right inferior frontal gyrus and left subcallosal cortex/nucleus accumbens were taken into account. In contrast, neither right inferior frontal gyrus nor left subcallosal cortex/nucleus accumbens regions correlated significantly with Learning Rate when atrophy in the other regions was partialled out (*p*-values ≥ 0.2).

## Discussion

By employing a combined approach of computational modeling and VBM analysis, we show that PD patients have a learning acquisition deficit that is associated with volumetric reductions in discrete fronto-striatal regions. This is the first time that such learning deficits in PD have been probed via structural imaging techniques and our findings fit well with the broader learning literature, whilst highlighting a novel approach in order to further characterize discrimination learning in PD.

The nature of learning assessed in the current study reflects the formation of stimulus-response associations, which are learnt through incorporating feedback via a trial-and-error approach. Impaired learning acquisition rates on discrimination tasks have been demonstrated behaviorally in PD patients (Czernecki et al., 2002; Myers et al., 2003; de Wit et al., 2011; Shiner et al., 2012) and also in neurocomputational models of PD (Moustafa et al., 2010). Furthermore, Shohamy and colleagues (2004, 2006) have shown that in PD the feedback learning deficit is relatively specific, as patients are impaired when required to learn associations on the basis of feedback, but equivalent to controls when observational learning of the same associations was required.

Our results further confirm a feedback-based learning acquisition deficit in mild, non-demented PD. Patients and controls were equivalent in their exploration parameters, with both showing a minimal amount of exploration. This would be expected given the nature of the task wherein subjects are not encouraged to modify their responses as the stimulus-response-outcome contingencies do not change. Nevertheless, it further validates the utility of our model that it was able to identify this effect. Results from the analysis of learning accuracy across blocks indicated that deficient learning in the PD patients was mostly driven by poorer performance later in the task. We did not find a difference in explicit knowledge of stimulus: response-outcome contingencies, suggesting that despite a deficient learning rate the PD patients were ultimately able to attain a good level of knowledge of these contingencies (see also de Wit et al., 2011). The acquisition impairment did not correlate with any clinical disease variables; nor was a correlational relationship evident between learning rate and working memory (as assessed via the digit span backwards task), which was found to be mildly impaired. Importantly, on other executive domains assessed in the current study, the PD patients' performance was equivalent to controls, which supports the notion of a discrete discrimination learning deficit in this patient group.

The previous findings relating deficient feedback-based learning in PD to dopamine dysfunction have been somewhat equivocal, as comparisons between patients ON vs. OFF medication have found that performance on a variety of learning tasks is impaired in both scenarios (Czernecki et al., 2002; Ell et al., 2010; Moustafa and Gluck, 2011), or that performance differs based on task demands (Shohamy et al., 2006) or valence of feedback signals (Frank et al., 2004). A number of studies using feedback-based category learning in PD have suggested that respective demands on selective attention vs. working memory, which are differentially affected by dopamine therapy, may determine learning performance (Filoteo et al., 2005, 2007). Given that in the OFF state patients suffer severe depletion in dorsal striatum and its projection targets, whilst the ON state is associated with restoration of those levels and the possibility of dopaminergic “overdose” in ventral striatum and limbic regions (Cools et al., 2001), differential effects on discrimination learning would be expected. Nonetheless, the finding of similar effects arising from two ostensibly disparate conditions has been explained with respect to the “relative” rather than “absolute” levels of dopamine, as a reduced dynamic range of phasic dopamine activity can result from both the ON and OFF states (Frank, 2005).

In contrast to previous studies that have characterized discrimination learning deficits in PD with respect to dopaminergic dysfunction, our current results define these deficits with respect to the possible structural abnormalities that may be contributory. In addition to dopamine depletion, PD is also associated with gray matter loss and synaptic denervation in fronto-striatal regions essential to broad aspects of learning and feedback processing, including the striatum (Rosenberg-Katz et al., 2013), medial temporal regions (Filoteo et al., 2013) and ventromedial prefrontal cortex (O'Callaghan et al., 2013). More specifically, prefrontal volume loss has been identified in non-demented PD, in comparison to healthy controls (Song et al., 2011; Melzer et al., 2012). Our findings reveal that discrete fronto-striatal regions, namely ventromedial prefrontal cortex, right inferior frontal gyrus and nucleus accumbens, are directly associated with acquisition deficits during feedback-based discrimination learning. The presence of underlying gray matter loss contributing to learning deficits may to some degree explain why discrimination learning can be affected both ON and OFF medication, and thus indicate that dopamine imbalance may not be the sole explanation for learning deficits in PD.

Our findings potentially shed light on previous reports that disease severity in PD is associated with specific learning impairments (Owen et al., 1993; Swainson et al., 2006). In particular, Swainson et al. (2006) found that early-stage, unmedicated patients were not impaired on a complex discrimination learning task; whilst early-stage, medicated patients *were* impaired on the task, their performance was mediated by deficient perceptual categorization of the complex stimuli, rather than a learning deficit *per se*. In contrast, only patients with severe, medicated PD showed impaired learning in the absence of perceptual categorization deficits. This raises the possibility that some factor other than inappropriate dopamine levels may intervene in later-stage PD to produce learning impairments on the task. Interestingly, the comparison groups of Huntington's disease and frontal lobe lesion patients included in the study showed the same pattern of intact perceptual categorization, but impaired learning, suggesting that more extensive fronto-striatal dysfunction may underpin the learning impairments. Taken together with our findings, it may be that fronto-striatal atrophy is a contributing factor to those learning impairments seen in PD with disease progression.

The possibility that fronto-striatal atrophy can mediate learning performance is also relevant to previous studies that have identified considerable variation within their PD cohorts. For example, using a rule-based category learning task, Ashby et al. (2003) found that PD patients were impaired at the group level, however, this effect was driven by impaired performance in only half of the patients, with the remainder performing equivalent to controls. The authors interpreted this as evidence of distinctive PD sub-groups. Indeed, differences in the clinical phenotypes of PD are well recognized (Lewis et al., 2005) and evidence is accumulating that the presence of more widespread fronto-subcortical atrophy may be characteristic of certain sub-groups (Feldmann et al., 2008; Melzer et al., 2012; Rosenberg-Katz et al., 2013). An admixture of PD patients with and without prefrontal volume loss may contribute to within-group variation in learning performance.

Results from our partial correlation analysis suggest that atrophy in the ventromedial prefrontal region may be driving the association with acquisition deficits. Although previous research using functional MRI in healthy controls has identified striatal activity as crucial during the acquisition phase of learning tasks (Pessiglione et al., 2006; Foerde and Shohamy, 2011a), others have shown ventromedial prefrontal cortex activity during learning acquisition (de Wit et al., 2009). Whereas the gradual learning of stimulus-response associations is presumed to reflect “habit” learning that is mediated by basal ganglia dopamine signals (Shohamy et al., 2008), “goal-directed” learning, which involves a focus on stimulus-response-outcome associations, has been linked to medial prefrontal regions (Balleine and O'Doherty, 2009). The interplay between the habitual and goal-directed modes can be explained by the “dual-systems” account, whereby instrumental learning can be supported by either modality (Dickinson and Balleine, 1994; de Wit and Dickinson, 2009). In line with the possibility that acquisition of instrumental discriminations is partly supported by goal-directed learning, de Wit et al. (2009) showed that engagement of the ventromedial prefrontal cortex during discrimination learning was predictive of goal-directed performance during a subsequent test phase. During that “instructed outcome-devaluation” test phase, participants were told that some of the fruit outcomes were no longer worth points. Participants with relatively strong engagement of the ventromedial prefrontal cortex during learning were better able to direct their responses toward the still-valuable outcomes and away from the devalued ones. More recently, individual differences in the strength of the white-matter pathway between the ventromedial prefrontal cortex and caudate have also been implicated in goal-directed control, whilst connectivity between the posterior putamen and premotor cortex has been related to habit learning (de Wit et al., 2012). Given these previous investigations of the role of the ventromedial prefrontal cortex in action control, our results are in keeping with a deficit in goal-directed learning.

In the category learning literature, the Competition between Verbal and Implicit Systems model (COVIS; Ashby et al., 1998) has been proposed to explain the neural systems that mediate rule-based learning vs. procedural (information-integration) learning. Whilst both are inherently feedback-based, these learning mechanisms necessitate different strategies and depend on divergent systems. The former comprising of an explicit hypothesis-testing system underpinned by a broad network including prefrontal cortex, anterior cingulate, hippocampus and caudate head; and the latter, requiring perceptual information to be integrated at a pre-decisional level, is mediated by cortical-striatal synapses within the putamen and premotor cortex circuitry (Ashby and Maddox, 2011). However, there is growing consensus that prefrontal regions, in particular ventromedial prefrontal cortex, may play a role in both types of learning (Seger, 2008). Schnyer et al. (2009) explored this directly by contrasting ventromedial prefrontal cortex lesion patients on rule-based vs. information-integration learning and found that patients were impaired in both types of learning. Work by Seger and colleagues (Seger and Cincotta, 2005; Seger et al., 2010) has also highlighted the role of the ventral striatum in encoding feedback during unstructured category learning tasks. These findings suggest that the ventromedial prefrontal cortex and ventral striatum—important hubs in the cortico-striatal motivational loop—are critical for monitoring and integrating feedback, regardless of the learning strategy.

Ventromedial prefrontal regions and ventral striatum (particularly nucleus accumbens) are also more generally associated with reward processing (Kringelbach, 2005), which may further explain why these regions were implicated in acquisition learning deficits in our patients, as the feedback involved in the task was reward-oriented. Specific reward-learning deficits have previously been demonstrated in PD (Swainson et al., 2000; Housden et al., 2010), and based on the volumetric reductions we found in regions crucial to reward processing in our patient cohort, it is likely that deficient reward processing may have contributed to the acquisition deficits. Our finding that the right inferior frontal gyrus was also associated with the acquisition deficit may reflect the demands of more general cognitive control that is required in such a learning task. The right inferior frontal gyrus is well known to be implicated in inhibitory control of behavior (Aron et al., 2004), however, a broader interpretation of its action is that it is involved in the detection/monitoring of task-relevant cues (Hampshire et al., 2010) and in terms of learning processes, the region is recruited during reversal learning (Cools et al., 2002).

From a mechanistic account, the involvement of prefrontal regions in learning from trial-by-trial feedback is also emphasized in computational models that seek to integrate basal ganglia and prefrontal function with respect to higher level executive processes. In the computational accounts proposed by O'Reilly and Frank (2006), the prefrontal cortex is active in maintaining information, whereby task-relevant information is determined via basal ganglia-prefrontal interactions that serve as a gating mechanism (see also Hazy et al., 2007). In these models, basal ganglia dopamine-dependant learning systems are presumed to trigger updates of working memory representations in the prefrontal cortex, whilst simultaneously inhibiting task-irrelevant information—thus allowing intrinsic prefrontal cortical mechanisms to actively maintain the contents of working memory. Our results suggest that direct atrophy in prefrontal regions may interfere with the updating and maintenance of task-relevant information in these models, which may therefore contribute to deficient acquisition on learning tasks.

The VBM technique utilized in this study is not without limitations, including registration and normalization issues and imperfect gray-white matter segmentation, particularly in relation to already atypical brains (Mechelli et al., 2005). In addition, the analysis we conducted does not measure the particular morphological changes brain structures undergo in PD and in interpreting findings of reduced gray matter density, it must be borne in mind that the precise mechanisms of cell degeneration in PD are still a matter of debate (Obeso et al., 2010). Nevertheless, VBM provides an important tool to further characterize learning systems in PD.

Together, our findings suggest that discrete fronto-striatal regions contribute to the feedback-based learning deficits in PD. It is likely that gray matter loss in these regions interacts with dopaminergic dysfunction to produce these deficits, and that the ultimate behavioral manifestation reflects an interplay between neurotransmitter imbalance and underlying structural changes. Our findings have important implications for the development of learning theories based on PD as a model of dopaminergic dysfunction. Whereby current theories and computational approaches have tended to focus on dopamine imbalance in intra-basal ganglia circuitry, a broader appreciation of the more distributed brain changes, such as gray matter loss, and how these may also affect learning processes is crucial in order to continue to refine these theoretical models. These results highlight that dysfunction in dopaminergic systems may not be the sole explanation for feedback-based learning deficits in PD, but that gray matter loss may also contribute to these deficits.

## Author contributions

Claire O'Callaghan contributed to the design and conceptualization of the study, data collection, analysis and interpretation of data, drafting and revision of the manuscript. Ahmed A. Moustafa carried out the computational modeling and contributed to interpretation of the data and revision of the manuscript. Sanne de Wit contributed to study conceptualization and manuscript revision. James M. Shine contributed to data collection and manuscript revision. Trevor W. Robbins was involved in revision of the manuscript. Simon J. G. Lewis contributed to interpretation of the data and revision of the manuscript. Michael Hornberger contributed to design and conceptualization of the study, analysis and interpretation of data, and revision of the manuscript.

### Conflict of interest statement

The authors declare that the research was conducted in the absence of any commercial or financial relationships that could be construed as a potential conflict of interest.
