# Supplementary material for: Fronto-striatal gray matter contributions to discrimination learning in Parkinson's disease
Source: Front Comput Neurosci. 2013 Dec 12;7:180. doi: 10.3389/fncom.2013.00180 (PMC3859902; doi:10.3389/fncom.2013.00180)
Supplement: Supplementary file 1 [file DataSheet1.DOC]

**Supplementary Table 1**. Region of interest Voxel-based morphometry (VBM) results showing regions of significant grey matter intensity decrease for the PD group in comparison to Controls. All results corrected for multiple comparisons (FWE) at *p* < .05.

| ***Regions*** | ***Hemisphere (L/R/B)*** | ***MNI coordinates***  ***X Y Z*** | | | ***Number of voxels*** | ***T score*** |
| --- | --- | --- | --- | --- | --- | --- |
| **PD vs. Controls** |  |  |  |  |  |  |
| Frontal orbital cortex; Inferior frontal gyrus | R | 22 | 30 | -22 | 939 | 2.05 |
| Frontal medial/Subcallosal cortices; Left Nucleus accumbens  Inferior frontal gyrus | B  L | -6  -54 | 26  32 | -18  -2 | 808  322 |  |
